# Supplementary material for: Mutations in PCYT2 disrupt etherlipid biosynthesis and cause a complex hereditary spastic paraplegia
Source: Brain. 2019 Oct 22;142(11):3382–97. doi: 10.1093/brain/awz291 (PMC6821184; doi:10.1093/brain/awz291)
Supplement: awz291_Supplementary_Data [file awz291_supplementary_data.zip › awz291-Suppl_data/Supplementary_Data4.zip!/Supplementary information 3 - fibroblast lipidomics.zip!/Fibroblast lipidomics/Controle vs Patient1/Statistical plots and data/LPC[O] species/Heatmap.pdf]

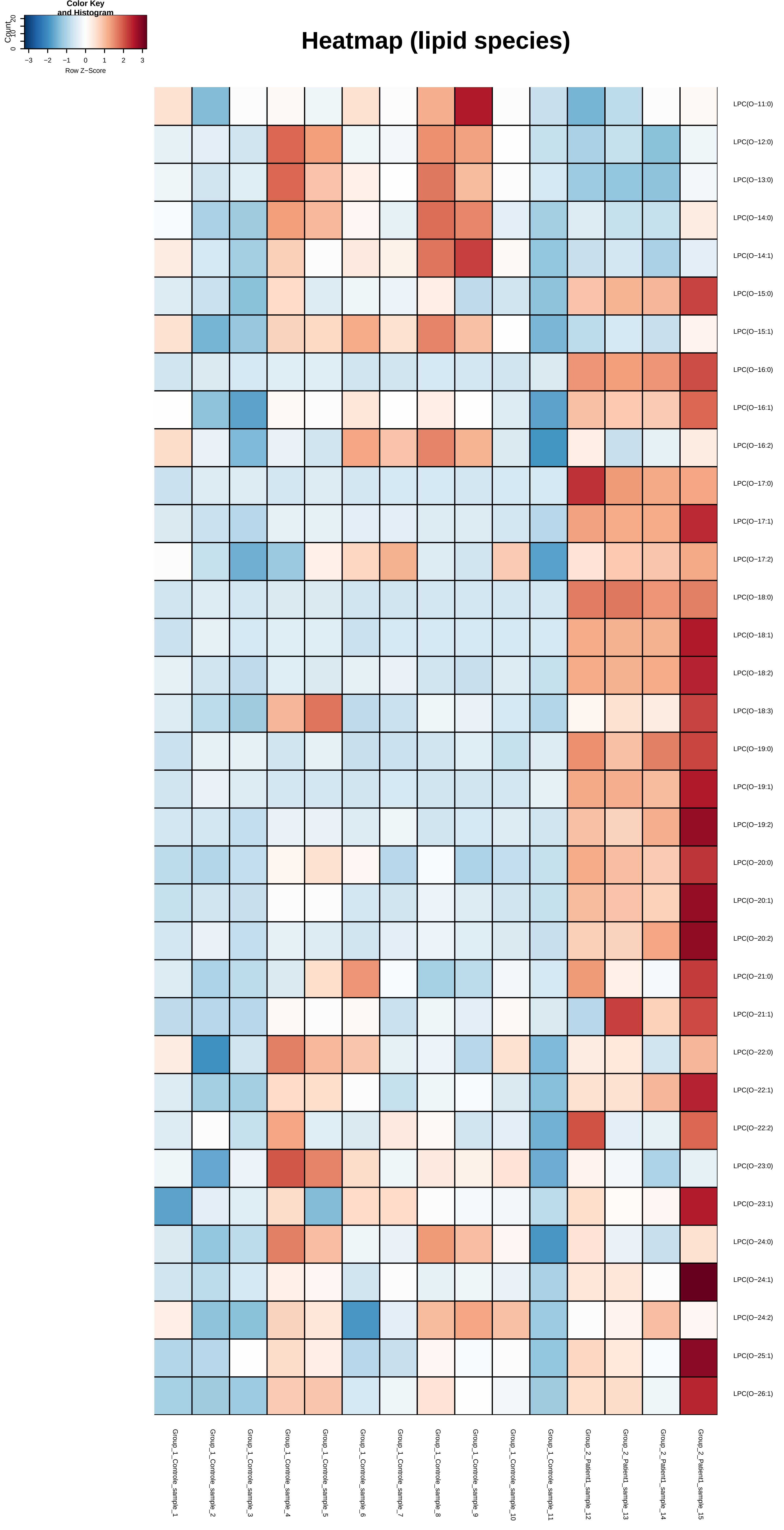
Color Key
and Histogram
Count
0
10
20
−3
−2
−1
0
1
2
3
Row Z−Score
Heatmap (lipid species)
LPC(O−11:0)
LPC(O−12:0)
LPC(O−13:0)
LPC(O−14:0)
LPC(O−14:1)
LPC(O−15:0)
LPC(O−15:1)
LPC(O−16:0)
LPC(O−16:1)
LPC(O−16:2)
LPC(O−17:0)
LPC(O−17:1)
LPC(O−17:2)
LPC(O−18:0)
LPC(O−18:1)
LPC(O−18:2)
LPC(O−18:3)
LPC(O−19:0)
LPC(O−19:1)
LPC(O−19:2)
LPC(O−20:0)
LPC(O−20:1)
LPC(O−20:2)
LPC(O−21:0)
LPC(O−21:1)
LPC(O−22:0)
LPC(O−22:1)
LPC(O−22:2)
LPC(O−23:0)
LPC(O−23:1)
LPC(O−24:0)
LPC(O−24:1)
LPC(O−24:2)
LPC(O−25:1)
LPC(O−26:1)
Group_1_Controle_sample_1
Group_1_Controle_sample_2
Group_1_Controle_sample_3
Group_1_Controle_sample_4
Group_1_Controle_sample_5
Group_1_Controle_sample_6
Group_1_Controle_sample_7
Group_1_Controle_sample_8
Group_1_Controle_sample_9
Group_1_Controle_sample_10
Group_1_Controle_sample_11
Group_2_Patient1_sample_12
Group_2_Patient1_sample_13
Group_2_Patient1_sample_14
Group_2_Patient1_sample_15
